# 3-(10-Chloro-9-anthr­yl)-5-[3-(prop-2-yn­yloxy)phenoxy­meth­yl]isoxazole

**DOI:** 10.1107/S1600536809019229

**Published:** 2009-05-29

**Authors:** Juan Li, Hailing Xi, Jianming Zhang

**Affiliations:** a Key Laboratory of Pesticide and Chemical Biology of the Ministry of Education, College of Chemistry, Central China Normal University, Wuhan 430079, People’s Republic of China; b 6th Department, Research Institute of Chemical Defence, Beijing 102205, People’s Republic of China

## Abstract

In the title mol­ecule, C_27_H_18_ClNO_3_, the anthracene mean plane forms dihedral angles of 67.43 (2) and 15.75 (3)° with the isoxazole and benzene rings, respectively. In the crystal structure, C—H⋯π inter­actions link mol­ecules into centrosymmetric dimers, which are further linked by weak inter­molecular C—H⋯N hydrogen bonds into ribbons propagating in the [110] direction.

## Related literature

For the preparation of the title compound, see Han *et al.* (2003 ▶). For pharmaceutical applications of isoxazole and its derivatives, see: De Luca *et al.* (2001 ▶); Yamamoto *et al.* (2007 ▶); Reuman *et al.* (2008 ▶).
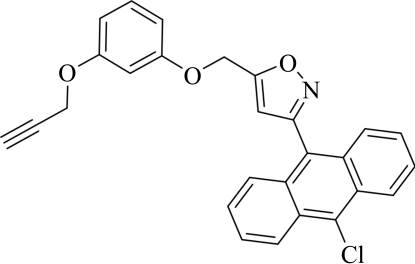


## Experimental

### 

#### Crystal data

C_27_H_18_ClNO_3_
*M* *_r_* = 439.87
Triclinic, 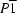

*a* = 8.4816 (3) Å
*b* = 8.6450 (3) Å
*c* = 16.8606 (6) Å
α = 100.364 (1)°
β = 103.596 (1)°
γ = 94.965 (1)°
*V* = 1171.25 (7) Å^3^
*Z* = 2
Mo *K*α radiation
μ = 0.19 mm^−1^
*T* = 292 K
0.30 × 0.20 × 0.10 mm

#### Data collection

Bruker SMART 4K CCD area-detector diffractometer
Absorption correction: none
4812 measured reflections
4812 independent reflections
4227 reflections with *I* > 2σ(*I*)
*R*_int_ = 0.0000

#### Refinement

*R*[*F* ^2^ > 2σ(*F* ^2^)] = 0.057
*wR*(*F* ^2^) = 0.163
*S* = 1.06
4812 reflections
289 parameters
H-atom parameters constrained
Δρ_max_ = 0.32 e Å^−3^
Δρ_min_ = −0.39 e Å^−3^

### 

Data collection: *SMART* (Bruker, 2001 ▶); cell refinement: *SAINT* (Bruker, 2001 ▶); data reduction: *SAINT*; program(s) used to solve structure: *SHELXS97* (Sheldrick, 2008 ▶); program(s) used to refine structure: *SHELXL97* (Sheldrick, 2008 ▶); molecular graphics: *SHELXTL* (Sheldrick, 2008 ▶); software used to prepare material for publication: *SHELXTL* and *PLATON* (Spek, 2009 ▶).

## Supplementary Material

Crystal structure: contains datablocks I, global. DOI: 10.1107/S1600536809019229/cv2557sup1.cif

Structure factors: contains datablocks I. DOI: 10.1107/S1600536809019229/cv2557Isup2.hkl

Additional supplementary materials:  crystallographic information; 3D view; checkCIF report

## Figures and Tables

**Table 1:** Hydrogen-bond geometry (Å, °) *Cg* is the centroid of the C19–C24 ring.

*D*—H⋯*A*	*D*—H	H⋯*A*	*D*⋯*A*	*D*—H⋯*A*
C27—H27⋯N1^i^	0.93	2.57	3.465 (3)	163
C16—H16⋯*Cg*^ii^	0.93	2.67	3.431 (2)	140

Symmetry codes: (i) 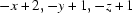; (ii) 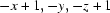.

**Fig. 1.**
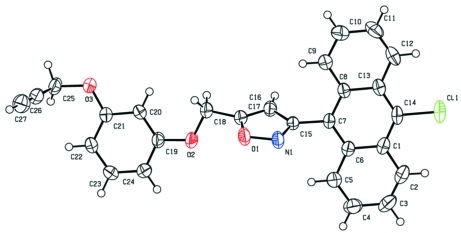
View of the molecule of (I) showing the atom-labelling scheme. Displacement ellipsoids are drawn at the 50% probability level. H atoms are represented by spheres of arbitrary radius.

## supplementary crystallographic information

### Comment

Isoxazole and isoxazole derivatives are important pharmaceutical agents (De Luca *et al.*, 2001), so they are widely investigated (Yamamoto *et al.*, 2007; Reuman *et al.*, 2008). As a part of our investigation into isoxazole derivatives, we report here the structure of the title compound (I).

In (I) (Fig. 1), the anthracene mean plane forms the dihedral angles of 67.43 (2)° and 15.75 (3)° with the isoxazole and benzene rings, respectively. In the crystal, C—H···π interactions (Table 1) link the molecules into centrosymmetric dimers, which are further linked by the weak intermolecular C—H···N hydrogen bonds (Table 1) into ribbons propagated in direction [110]. The porous crystal packing contains voids of 171 Å^3^.

### Experimental

The title compound was synthesized according to the procedure of Han *et al.* (2003) in 32% isolated yield. Crystals of (I) suitable for X-ray data collection were obtained by slow evaporation of a methaoland DMF solution in ratio of 50:1 at 298 K.

### Refinement

All H atoms bonded to C atoms were initially located in difference Fourier maps and then constrained to their ideal geometry positions (*C*–*H* = 0.93-0.97 Å), and refined as riding with Uĩso~(H) = 1.2*U*~eq~(C).

### Crystal data

**Table:**

C_27_H_18_ClNO_3_	*Z* = 2
*M**_r_* = 439.87	*F*(000) = 456
Triclinic, *P*1	*D*_x_ = 1.247 Mg m^−^^3^
Hall symbol: -P 1	Mo *K*α radiation, λ = 0.71073 Å
*a* = 8.4816 (3) Å	Cell parameters from 6384 reflections
*b* = 8.6450 (3) Å	θ = 2.5–28.2°
*c* = 16.8606 (6) Å	µ = 0.19 mm^−^^1^
α = 100.364 (1)°	*T* = 292 K
β = 103.596 (1)°	Block, colourless
γ = 94.965 (1)°	0.30 × 0.20 × 0.10 mm
*V* = 1171.25 (7) Å^3^	

### Data collection

**Table:**

Bruker SMART 4K CCD area-detector diffractometer	4227 reflections with *I* > 2σ(*I*)
Radiation source: fine-focus sealed tube	*R*_int_ = 0.0000
graphite	θ_max_ = 26.5°, θ_min_ = 2.4°
φ and ω scans	*h* = −10→10
4812 measured reflections	*k* = −10→10
4812 independent reflections	*l* = 0→21

### Refinement

**Table:**

Refinement on *F*^2^	Primary atom site location: structure-invariant direct methods
Least-squares matrix: full	Secondary atom site location: difference Fourier map
*R*[*F*^2^ > 2σ(*F*^2^)] = 0.057	Hydrogen site location: inferred from neighbouring sites
*wR*(*F*^2^) = 0.163	H-atom parameters constrained
*S* = 1.06	*w* = 1/[σ^2^(*F*_o_^2^) + (0.0786*P*)^2^ + 0.2928*P*] where *P* = (*F*_o_^2^ + 2*F*_c_^2^)/3
4812 reflections	(Δ/σ)_max_ = 0.001
289 parameters	Δρ_max_ = 0.32 e Å^−^^3^
0 restraints	Δρ_min_ = −0.39 e Å^−^^3^

### Special details

**Table:**

Geometry. All e.s.d.'s (except the e.s.d. in the dihedral angle between two l.s. planes) are estimated using the full covariance matrix. The cell e.s.d.'s are taken into account individually in the estimation of e.s.d.'s in distances, angles and torsion angles; correlations between e.s.d.'s in cell parameters are only used when they are defined by crystal symmetry. An approximate (isotropic) treatment of cell e.s.d.'s is used for estimating e.s.d.'s involving l.s. planes.
Refinement. Refinement of *F*^2^ against ALL reflections. The weighted *R*-factor *wR* and goodness of fit *S* are based on *F*^2^, conventional *R*-factors *R* are based on *F*, with *F* set to zero for negative *F*^2^. The threshold expression of *F*^2^ > σ(*F*^2^) is used only for calculating *R*-factors(gt) *etc*. and is not relevant to the choice of reflections for refinement. *R*-factors based on *F*^2^ are statistically about twice as large as those based on *F*, and *R*- factors based on ALL data will be even larger.

### Fractional atomic coordinates and isotropic or equivalent isotropic displacement parameters (Å^2^)

**Table:**

	*x*	*y*	*z*	*U*_iso_*/*U*_eq_	
C1	0.2863 (2)	0.3620 (2)	0.91382 (11)	0.0372 (4)	
C2	0.1335 (3)	0.4195 (2)	0.91474 (14)	0.0487 (5)	
H2	0.0916	0.4184	0.9609	0.058*	
C3	0.0490 (3)	0.4751 (3)	0.85017 (16)	0.0566 (5)	
H3	−0.0495	0.5128	0.8526	0.068*	
C4	0.1087 (3)	0.4767 (3)	0.77859 (15)	0.0534 (5)	
H4	0.0487	0.5145	0.7341	0.064*	
C5	0.2529 (2)	0.4234 (2)	0.77469 (12)	0.0426 (4)	
H5	0.2899	0.4244	0.7270	0.051*	
C6	0.3493 (2)	0.36581 (19)	0.84180 (10)	0.0338 (4)	
C7	0.4989 (2)	0.30988 (19)	0.83910 (10)	0.0318 (3)	
C8	0.5881 (2)	0.2499 (2)	0.90563 (10)	0.0335 (4)	
C9	0.7423 (2)	0.1965 (2)	0.90562 (12)	0.0430 (4)	
H9	0.7856	0.2003	0.8601	0.052*	
C10	0.8275 (3)	0.1400 (3)	0.97066 (14)	0.0543 (5)	
H10	0.9282	0.1063	0.9693	0.065*	
C11	0.7643 (3)	0.1322 (3)	1.04012 (13)	0.0580 (6)	
H11	0.8229	0.0920	1.0840	0.070*	
C12	0.6195 (3)	0.1825 (3)	1.04358 (11)	0.0504 (5)	
H12	0.5801	0.1767	1.0901	0.060*	
C13	0.5254 (2)	0.2444 (2)	0.97749 (10)	0.0369 (4)	
C14	0.3769 (2)	0.3003 (2)	0.97840 (11)	0.0393 (4)	
C15	0.56449 (19)	0.3098 (2)	0.76469 (10)	0.0313 (3)	
C16	0.5831 (2)	0.1769 (2)	0.70657 (11)	0.0372 (4)	
H16	0.5563	0.0702	0.7063	0.045*	
C17	0.6479 (2)	0.2387 (2)	0.65203 (10)	0.0369 (4)	
C18	0.7037 (2)	0.1683 (3)	0.57805 (11)	0.0447 (4)	
H18A	0.8181	0.2071	0.5854	0.054*	
H18B	0.6918	0.0536	0.5705	0.054*	
C19	0.6541 (2)	0.1848 (2)	0.43448 (11)	0.0379 (4)	
C20	0.7742 (2)	0.0930 (2)	0.42197 (11)	0.0378 (4)	
H20	0.8285	0.0457	0.4640	0.045*	
C21	0.8133 (2)	0.0720 (2)	0.34504 (11)	0.0356 (4)	
C22	0.7331 (2)	0.1422 (2)	0.28241 (12)	0.0428 (4)	
H22	0.7601	0.1289	0.2315	0.051*	
C23	0.6120 (2)	0.2329 (2)	0.29685 (12)	0.0444 (4)	
H23	0.5571	0.2799	0.2549	0.053*	
C24	0.5710 (2)	0.2549 (2)	0.37219 (12)	0.0429 (4)	
H24	0.4891	0.3156	0.3810	0.051*	
C25	0.9797 (3)	−0.0505 (2)	0.26169 (12)	0.0444 (4)	
H25A	0.8811	−0.0784	0.2163	0.053*	
H25B	1.0408	−0.1403	0.2597	0.053*	
C26	1.0785 (2)	0.0859 (2)	0.24940 (12)	0.0447 (4)	
C27	1.1606 (3)	0.1948 (3)	0.24028 (15)	0.0581 (6)	
H27	1.2255	0.2809	0.2331	0.070*	
Cl1	0.29951 (8)	0.29325 (7)	1.06495 (3)	0.0617 (2)	
N1	0.6140 (2)	0.44298 (18)	0.74604 (9)	0.0423 (4)	
O1	0.66859 (17)	0.39855 (16)	0.67356 (8)	0.0448 (3)	
O2	0.60568 (17)	0.2133 (2)	0.50758 (8)	0.0520 (4)	
O3	0.93481 (17)	−0.02142 (17)	0.33887 (8)	0.0473 (3)	

### Atomic displacement parameters (Å^2^)

**Table:**

	*U*^11^	*U*^22^	*U*^33^	*U*^12^	*U*^13^	*U*^23^
C1	0.0398 (9)	0.0328 (8)	0.0409 (9)	0.0008 (7)	0.0202 (7)	0.0011 (7)
C2	0.0488 (11)	0.0461 (11)	0.0581 (12)	0.0079 (8)	0.0309 (9)	0.0046 (9)
C3	0.0447 (11)	0.0537 (12)	0.0799 (15)	0.0180 (9)	0.0302 (11)	0.0117 (11)
C4	0.0455 (11)	0.0538 (12)	0.0653 (13)	0.0155 (9)	0.0136 (10)	0.0202 (10)
C5	0.0425 (10)	0.0460 (10)	0.0436 (10)	0.0086 (8)	0.0149 (8)	0.0140 (8)
C6	0.0373 (8)	0.0313 (8)	0.0342 (8)	0.0027 (6)	0.0143 (7)	0.0045 (6)
C7	0.0358 (8)	0.0333 (8)	0.0279 (8)	0.0031 (6)	0.0137 (6)	0.0039 (6)
C8	0.0394 (8)	0.0344 (8)	0.0279 (8)	0.0032 (7)	0.0118 (7)	0.0060 (6)
C9	0.0427 (10)	0.0528 (11)	0.0389 (9)	0.0120 (8)	0.0144 (8)	0.0155 (8)
C10	0.0475 (11)	0.0664 (13)	0.0528 (12)	0.0179 (10)	0.0087 (9)	0.0231 (10)
C11	0.0634 (13)	0.0702 (14)	0.0412 (11)	0.0117 (11)	0.0027 (10)	0.0265 (10)
C12	0.0624 (13)	0.0605 (12)	0.0280 (9)	0.0016 (10)	0.0099 (8)	0.0139 (8)
C13	0.0448 (9)	0.0374 (9)	0.0270 (8)	−0.0022 (7)	0.0111 (7)	0.0044 (6)
C14	0.0503 (10)	0.0390 (9)	0.0302 (8)	−0.0030 (7)	0.0218 (7)	0.0007 (7)
C15	0.0296 (7)	0.0390 (9)	0.0278 (8)	0.0065 (6)	0.0101 (6)	0.0093 (6)
C16	0.0433 (9)	0.0375 (9)	0.0343 (9)	0.0070 (7)	0.0165 (7)	0.0070 (7)
C17	0.0393 (9)	0.0444 (10)	0.0300 (8)	0.0106 (7)	0.0117 (7)	0.0089 (7)
C18	0.0493 (10)	0.0601 (12)	0.0309 (9)	0.0190 (9)	0.0174 (8)	0.0106 (8)
C19	0.0392 (9)	0.0472 (10)	0.0286 (8)	0.0072 (7)	0.0124 (7)	0.0051 (7)
C20	0.0406 (9)	0.0464 (10)	0.0302 (8)	0.0099 (7)	0.0109 (7)	0.0131 (7)
C21	0.0376 (9)	0.0397 (9)	0.0333 (8)	0.0067 (7)	0.0143 (7)	0.0097 (7)
C22	0.0448 (10)	0.0568 (11)	0.0339 (9)	0.0111 (8)	0.0163 (7)	0.0175 (8)
C23	0.0453 (10)	0.0555 (11)	0.0389 (9)	0.0154 (8)	0.0110 (8)	0.0217 (8)
C24	0.0405 (9)	0.0523 (11)	0.0391 (9)	0.0161 (8)	0.0117 (8)	0.0110 (8)
C25	0.0513 (10)	0.0448 (10)	0.0430 (10)	0.0123 (8)	0.0252 (8)	0.0039 (8)
C26	0.0413 (9)	0.0553 (11)	0.0408 (10)	0.0135 (8)	0.0154 (8)	0.0089 (8)
C27	0.0500 (11)	0.0662 (14)	0.0636 (14)	0.0066 (10)	0.0203 (10)	0.0206 (11)
Cl1	0.0751 (4)	0.0780 (4)	0.0412 (3)	0.0068 (3)	0.0364 (3)	0.0093 (2)
N1	0.0581 (9)	0.0403 (8)	0.0368 (8)	0.0081 (7)	0.0263 (7)	0.0101 (6)
O1	0.0603 (8)	0.0456 (7)	0.0381 (7)	0.0079 (6)	0.0277 (6)	0.0131 (5)
O2	0.0505 (8)	0.0849 (10)	0.0285 (6)	0.0301 (7)	0.0174 (6)	0.0122 (6)
O3	0.0562 (8)	0.0576 (8)	0.0425 (7)	0.0274 (6)	0.0266 (6)	0.0192 (6)

### Geometric parameters (Å, °)

**Table:**

C1—C14	1.395 (3)	C15—C16	1.416 (2)
C1—C2	1.430 (3)	C16—C17	1.340 (2)
C1—C6	1.441 (2)	C16—H16	0.9300
C2—C3	1.345 (3)	C17—O1	1.349 (2)
C2—H2	0.9300	C17—C18	1.485 (2)
C3—C4	1.416 (3)	C18—O2	1.418 (2)
C3—H3	0.9300	C18—H18A	0.9700
C4—C5	1.355 (3)	C18—H18B	0.9700
C4—H4	0.9300	C19—C20	1.376 (3)
C5—C6	1.427 (2)	C19—O2	1.377 (2)
C5—H5	0.9300	C19—C24	1.386 (3)
C6—C7	1.404 (2)	C20—C21	1.398 (2)
C7—C8	1.406 (2)	C20—H20	0.9300
C7—C15	1.487 (2)	C21—O3	1.374 (2)
C8—C9	1.424 (3)	C21—C22	1.382 (2)
C8—C13	1.440 (2)	C22—C23	1.385 (3)
C9—C10	1.358 (3)	C22—H22	0.9300
C9—H9	0.9300	C23—C24	1.379 (3)
C10—C11	1.407 (3)	C23—H23	0.9300
C10—H10	0.9300	C24—H24	0.9300
C11—C12	1.348 (3)	C25—O3	1.426 (2)
C11—H11	0.9300	C25—C26	1.461 (3)
C12—C13	1.427 (3)	C25—H25A	0.9700
C12—H12	0.9300	C25—H25B	0.9700
C13—C14	1.390 (3)	C26—C27	1.176 (3)
C14—Cl1	1.7434 (17)	C27—H27	0.9300
C15—N1	1.308 (2)	N1—O1	1.4082 (19)
			
C14—C1—C2	123.58 (17)	C16—C15—C7	127.76 (15)
C14—C1—C6	118.09 (16)	C17—C16—C15	104.87 (16)
C2—C1—C6	118.33 (17)	C17—C16—H16	127.6
C3—C2—C1	121.36 (19)	C15—C16—H16	127.6
C3—C2—H2	119.3	C16—C17—O1	109.76 (16)
C1—C2—H2	119.3	C16—C17—C18	133.60 (18)
C2—C3—C4	120.68 (19)	O1—C17—C18	116.60 (16)
C2—C3—H3	119.7	O2—C18—C17	107.63 (15)
C4—C3—H3	119.7	O2—C18—H18A	110.2
C5—C4—C3	120.19 (19)	C17—C18—H18A	110.2
C5—C4—H4	119.9	O2—C18—H18B	110.2
C3—C4—H4	119.9	C17—C18—H18B	110.2
C4—C5—C6	121.70 (18)	H18A—C18—H18B	108.5
C4—C5—H5	119.1	C20—C19—O2	123.81 (16)
C6—C5—H5	119.1	C20—C19—C24	121.26 (16)
C7—C6—C5	122.69 (16)	O2—C19—C24	114.93 (16)
C7—C6—C1	119.58 (15)	C19—C20—C21	118.90 (16)
C5—C6—C1	117.71 (16)	C19—C20—H20	120.6
C6—C7—C8	120.95 (15)	C21—C20—H20	120.6
C6—C7—C15	120.45 (14)	O3—C21—C22	124.87 (16)
C8—C7—C15	118.59 (15)	O3—C21—C20	114.38 (15)
C7—C8—C9	122.18 (15)	C22—C21—C20	120.76 (17)
C7—C8—C13	119.87 (16)	C21—C22—C23	118.85 (17)
C9—C8—C13	117.94 (15)	C21—C22—H22	120.6
C10—C9—C8	121.44 (18)	C23—C22—H22	120.6
C10—C9—H9	119.3	C24—C23—C22	121.41 (16)
C8—C9—H9	119.3	C24—C23—H23	119.3
C9—C10—C11	120.4 (2)	C22—C23—H23	119.3
C9—C10—H10	119.8	C23—C24—C19	118.82 (17)
C11—C10—H10	119.8	C23—C24—H24	120.6
C12—C11—C10	120.54 (18)	C19—C24—H24	120.6
C12—C11—H11	119.7	O3—C25—C26	112.99 (15)
C10—C11—H11	119.7	O3—C25—H25A	109.0
C11—C12—C13	121.53 (18)	C26—C25—H25A	109.0
C11—C12—H12	119.2	O3—C25—H25B	109.0
C13—C12—H12	119.2	C26—C25—H25B	109.0
C14—C13—C12	123.94 (17)	H25A—C25—H25B	107.8
C14—C13—C8	117.93 (16)	C27—C26—C25	178.8 (2)
C12—C13—C8	118.13 (18)	C26—C27—H27	180.0
C13—C14—C1	123.58 (16)	C15—N1—O1	105.45 (14)
C13—C14—Cl1	118.27 (14)	C17—O1—N1	108.51 (13)
C1—C14—Cl1	118.15 (15)	C19—O2—C18	117.55 (14)
N1—C15—C16	111.40 (15)	C21—O3—C25	117.66 (14)
N1—C15—C7	120.84 (15)		

### Hydrogen-bond geometry (Å, °)

**Table:**

*D*—H···*A*	*D*—H	H···*A*	*D*···*A*	*D*—H···*A*
C27—H27···N1^i^	0.93	2.57	3.465 (3)	163
C16—H16···Cg^ii^	0.93	2.67	3.431 (2)	140

Symmetry codes: (i) −*x*+2, −*y*+1, −*z*+1; (ii) −*x*+1, −*y*, −*z*+1.
